# Supplementary material for: High-Dose Intermittent Treatment with the Multikinase Inhibitor Sunitinib Leads to High Intra-Tumor Drug Exposure in Patients with Advanced Solid Tumors
Source: Cancers (Basel). 2022 Dec 9;14(24):6061. doi: 10.3390/cancers14246061 (PMC9775433; doi:10.3390/cancers14246061)
Supplement: Supplementary file 1 [file cancers-14-06061-s001.zip › cancers-2032617-SI/Supplementary Data S1.pdf]

Supplementary Data S1:

Relationship between:
sunitinib + and N-desethyl sunitinib (SUM) plasma-average or plasma-trough and corresponding tumor- and skin concentrations.

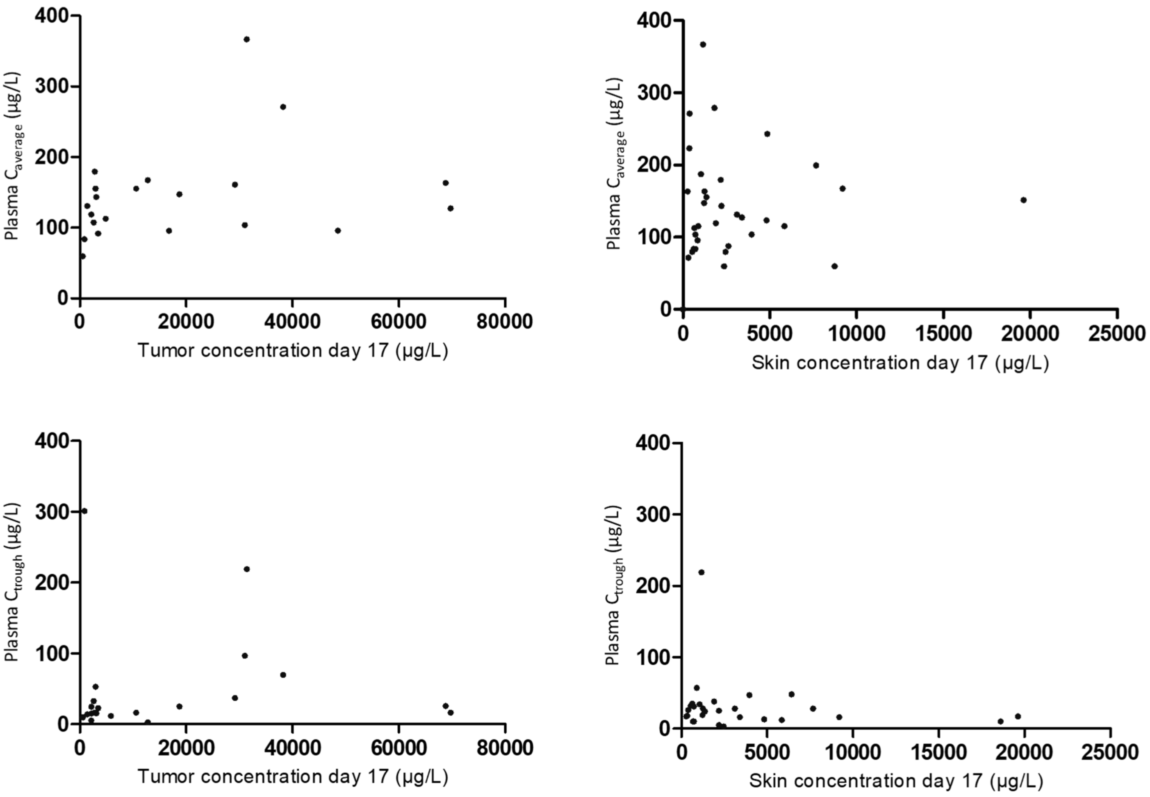

Supplementary Data S1:

Effect of food on the pharmacokinetics of high-dose intermittent sunitinib + N-desethyl sunitinib (SUM) concentration.

An extra cohort was opened to evaluate the effect of food on the interpatient variation in bioavailability and to study whether concomitant intake with food could further increase the maximum plasma concentration (C_{max}). Patients were randomized to either start 700 mg sunitinib after a 10-hour fasted regimen or within 30 minutes after a high-fat, high-calorie meal, followed by the alternative intake regimen at the second cycle, 2 weeks later. Subsequently patients continued the high-dose intermittent sunitinib once every 2 weeks regardless of food. Geometric mean ratios (GMR) and 90% confidence intervals (CI) of the sum concentration of sunitinib and SU12662 were estimated.

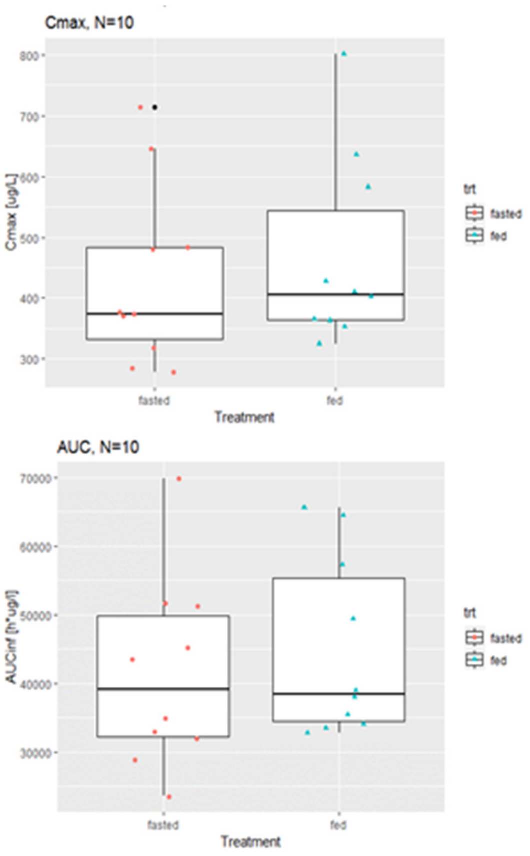

Sunitinib + N-desethyl sunitinib pharmacokinetic parameters (including 95% CI) for 10 patients receiving sunitinib 700 mg Q2W with (fed) and without (fasted) food.

The effect of food and the appearance of vomiting and/or diarrhea within 6 hours after sunitinib ingestion, were tested as a binary covariate on relative bioavailability and absorption rate, but did not statistically improve model fit.
