# Supplementary material for: High-Dose Intermittent Treatment with the Multikinase Inhibitor Sunitinib Leads to High Intra-Tumor Drug Exposure in Patients with Advanced Solid Tumors
Source: Cancers (Basel). 2022 Dec 9;14(24):6061. doi: 10.3390/cancers14246061 (PMC9775433; doi:10.3390/cancers14246061)
Supplement: Supplementary file 1 [file cancers-14-06061-s001.zip › cancers-2032617-SI/Supplementary Data S2.pdf]

**Supplementary data S2 – Methods (Details)**

Drug concentration measurements: Sunitinib and N-desethylsunitinib was measured in all samples; using a validated liquid chromatography–tandem mass spectrometry (LC-MS-MS). The system consisted of an Acquity H-Class UPLC system, coupled to a Xevo TQ-S micro tandem mass spectrometer. Sunitinib and N-desethyl sunitinib were separated on Waters Acquity UPLC BEH C18 column (2.1 × 100 millimeter (mm), 1.7 micrometer (μm) particle size) and eluted with a gradient elution system with a lower limit of detection of 2 μg/L for sunitinib and 5 μg/L for N-desethyl sunitinib and an upper limit of detection of 2000 μg/L for sunitinib and 5000 μg/L for N-desethyl sunitinib. Additionally, 10-fold dilution integrity was tested.

Immunohistochemistry: The following primary antibodies were used: CD3 (RM-9107-S; Thermo Fisher), CD31 (M082301, DAKO), M30 (PAS-16358, Thermo Fisher) and Ki-67 (M7240; Dako) and applied at dilutions of 1:320, 1:100, 1:200 and 1:25, respectively. Next, the slides were incubated for 30 minutes (min) with the appropriate secondary biotinylated antibody, followed by incubation with strep-ABC-HRP for 30 min. Finally, staining was visualized with 3,3-diamino-benzidine-tetra hydrochloride (DAB), 0.3 mg/ml in 1 ml PBS with 0.3 % H2O2. All slides were counterstained with hematoxylin.
