# Supplementary material for: High-Dose Intermittent Treatment with the Multikinase Inhibitor Sunitinib Leads to High Intra-Tumor Drug Exposure in Patients with Advanced Solid Tumors
Source: Cancers (Basel). 2022 Dec 9;14(24):6061. doi: 10.3390/cancers14246061 (PMC9775433; doi:10.3390/cancers14246061)

Supplementary data S3: Goodness-of-fit plots

A: Sunitinib – Observed versus population predicted

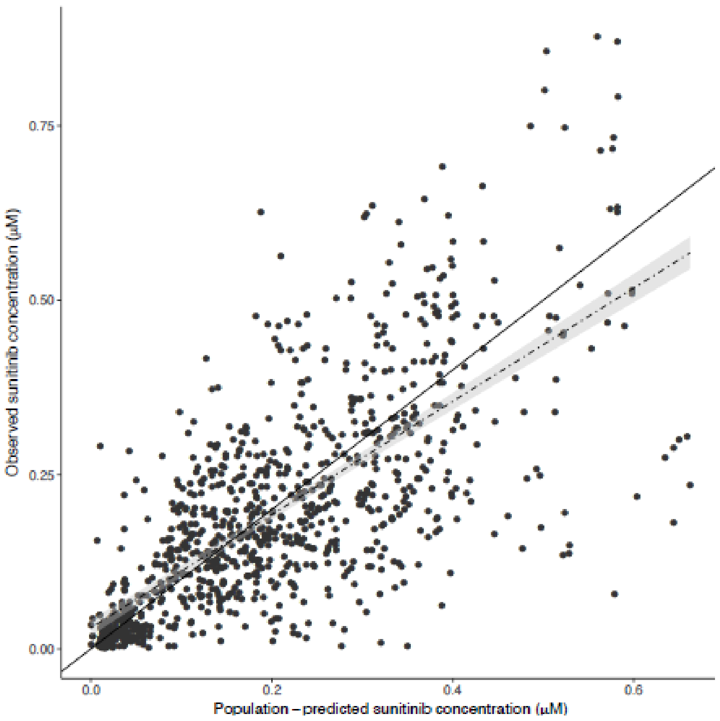

B: N-Desethylsunitinib - Observed versus population predicted

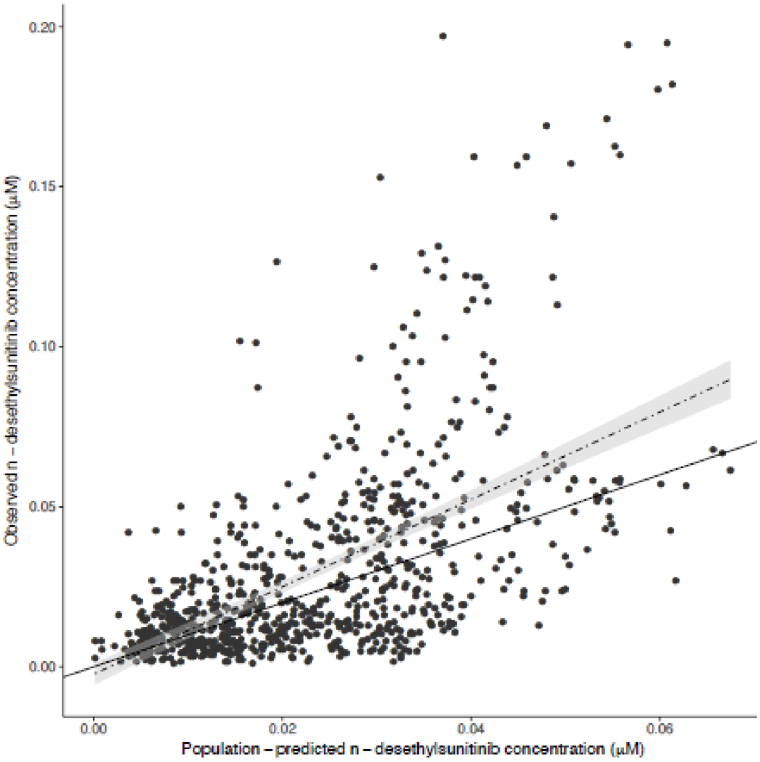

C: Sunitinib – Observed versus individually predicted

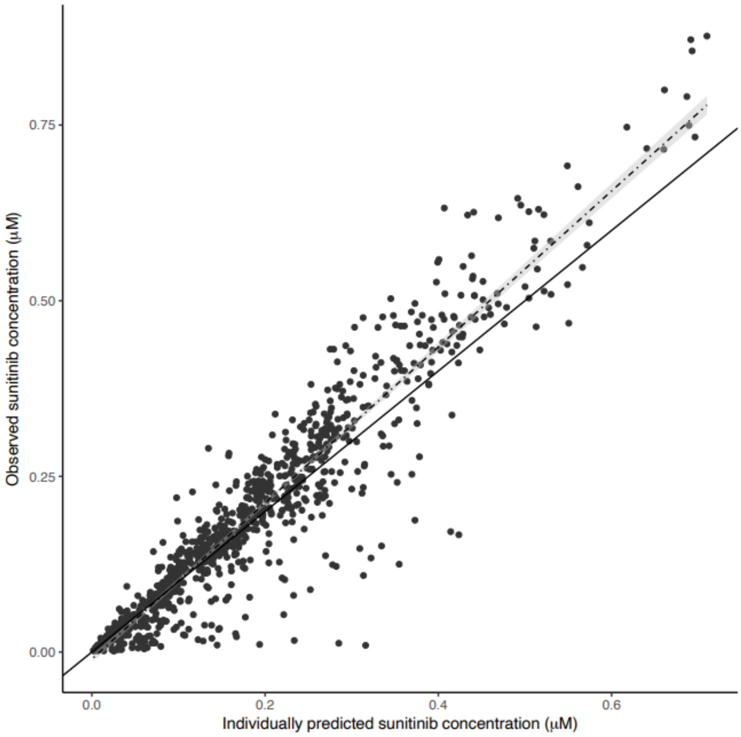

D: N-Desethylsunitinib – Observed versus individually predicted

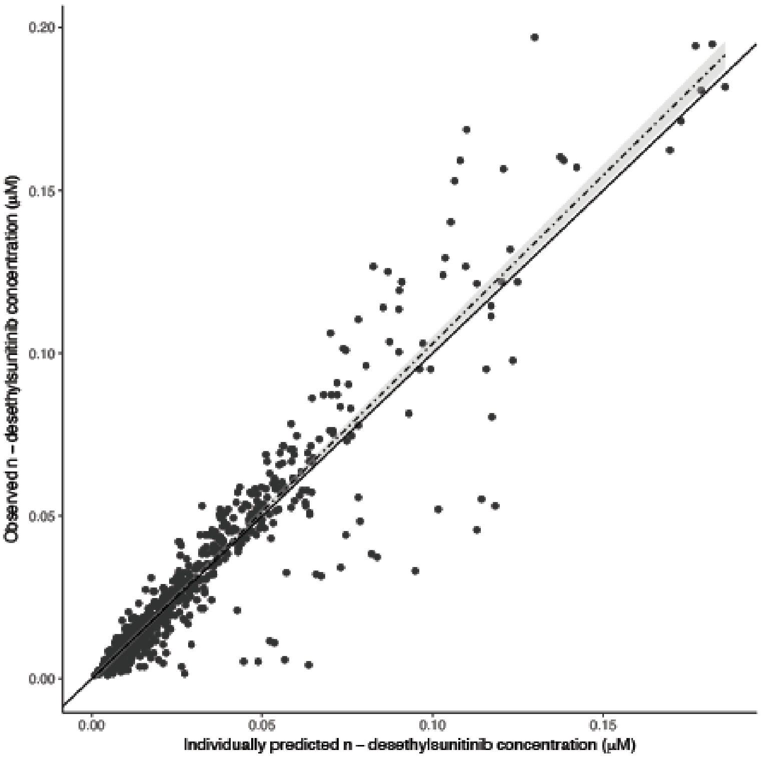

E: Sunitinib - CWRES versus population-predicted

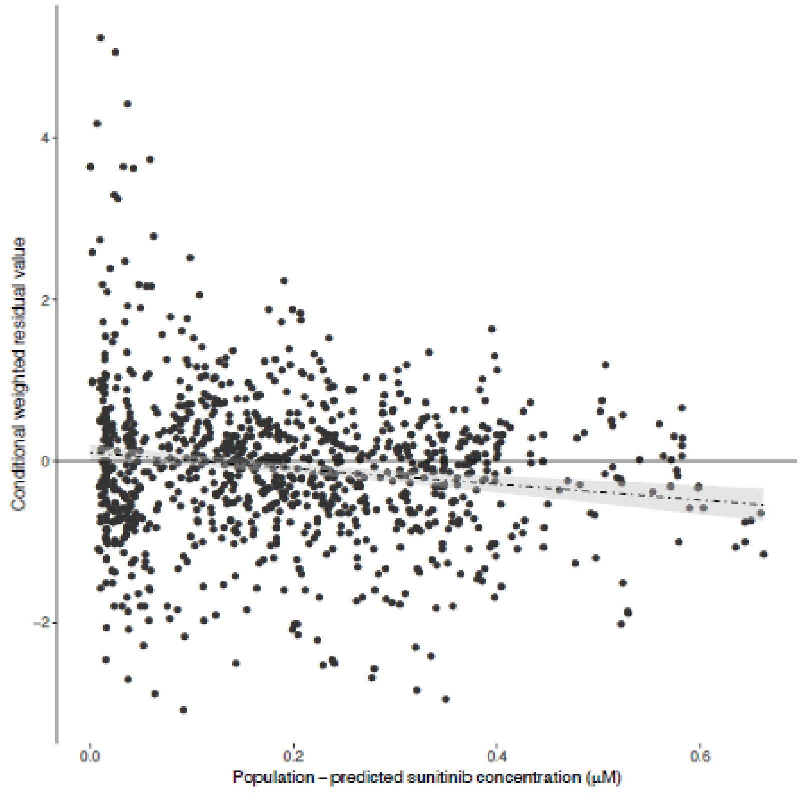

F: N-Desethylsunitinib - CWRES versus population-predicted

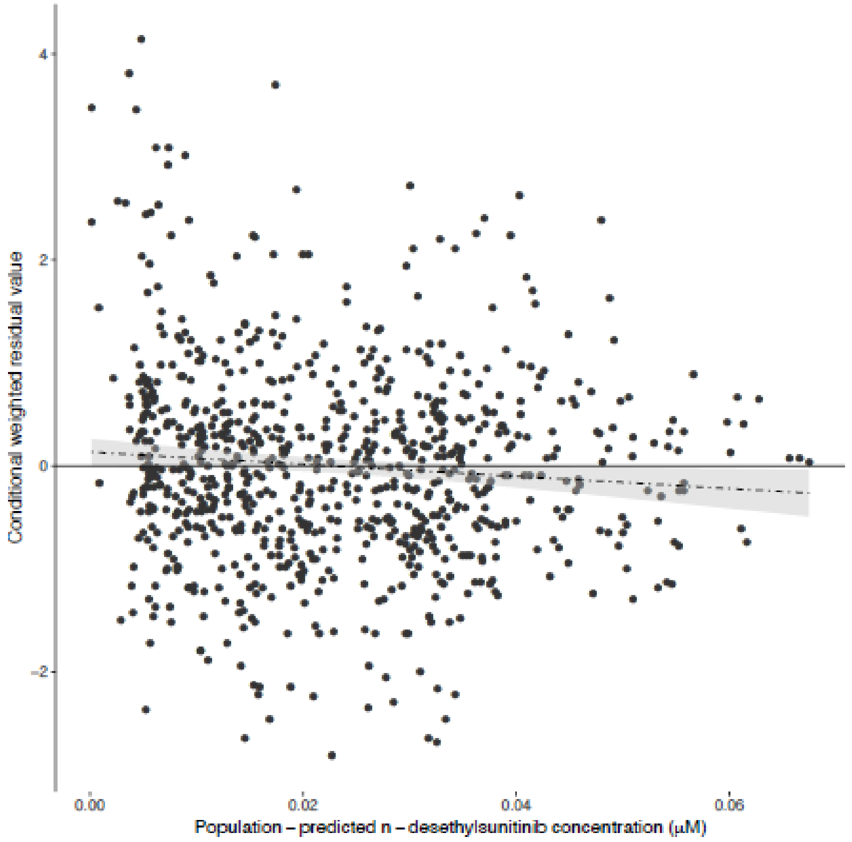

G: Sunitinib – CWRES versus Time after Dose

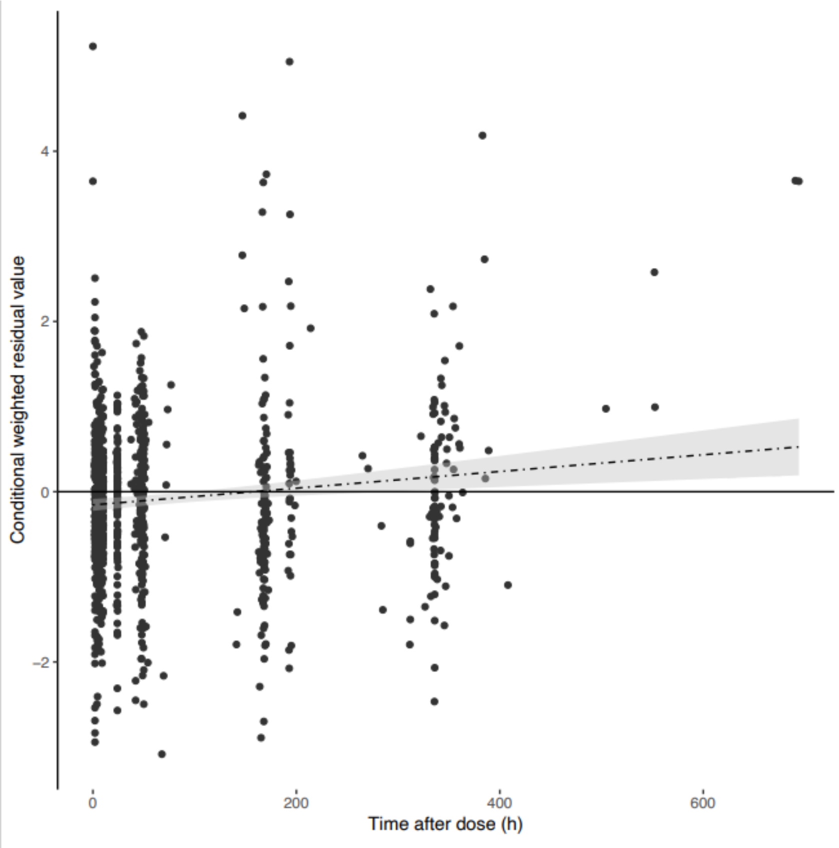

F: N-Desethylsunitinib – CWRES versus Time after Dose

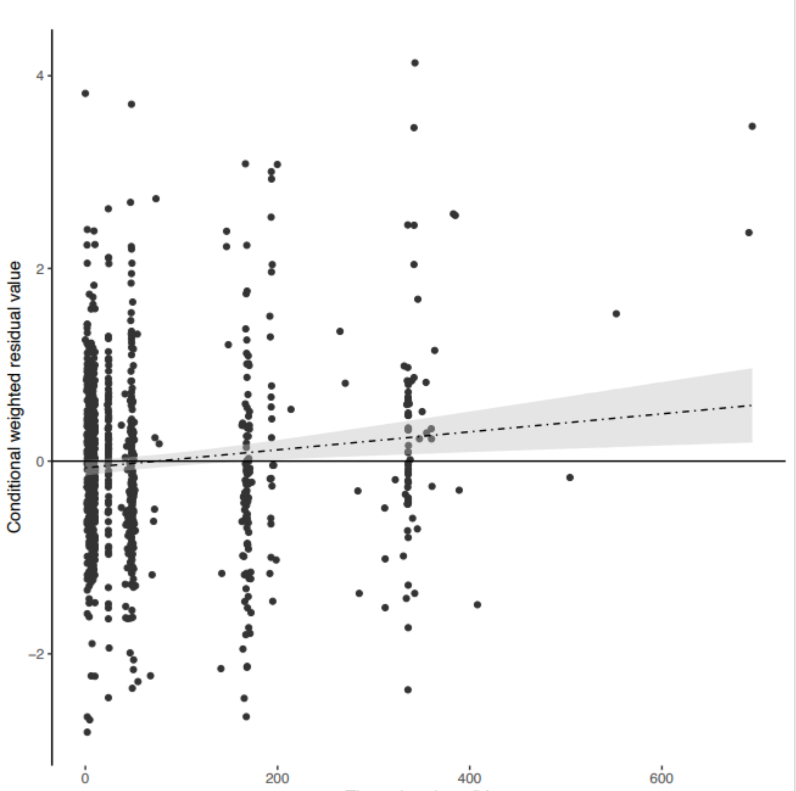

H: Sunitinib

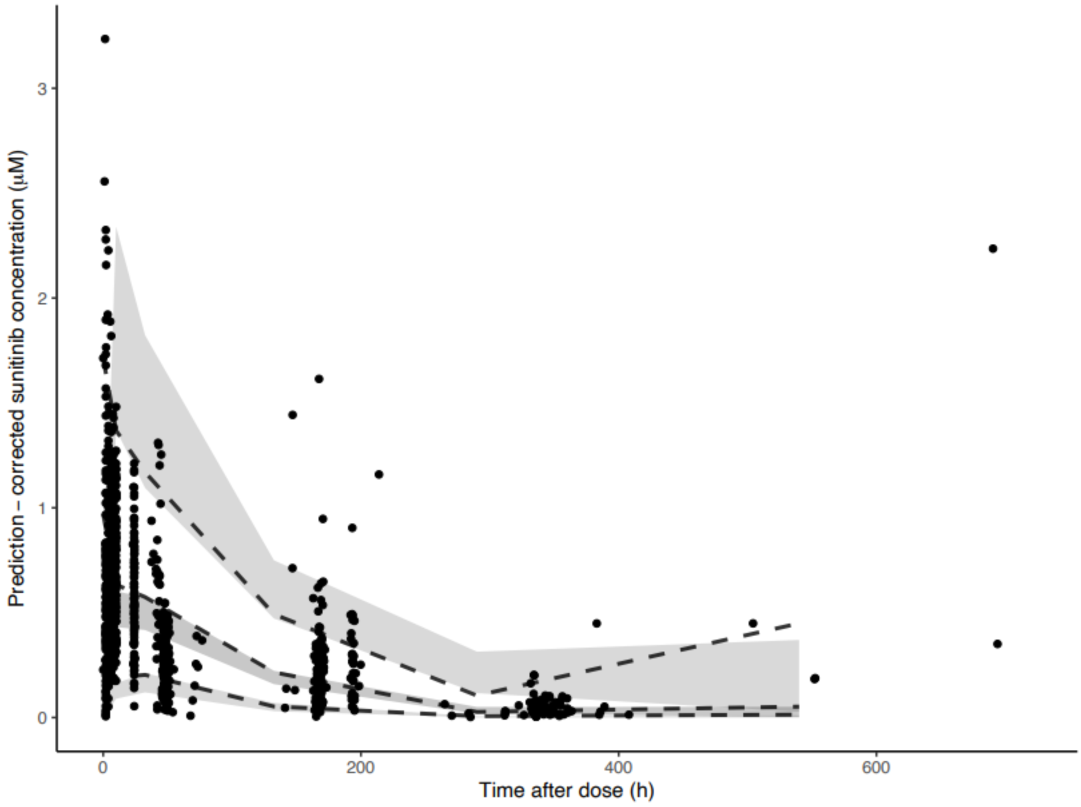

I: N-Desethylsunitinib

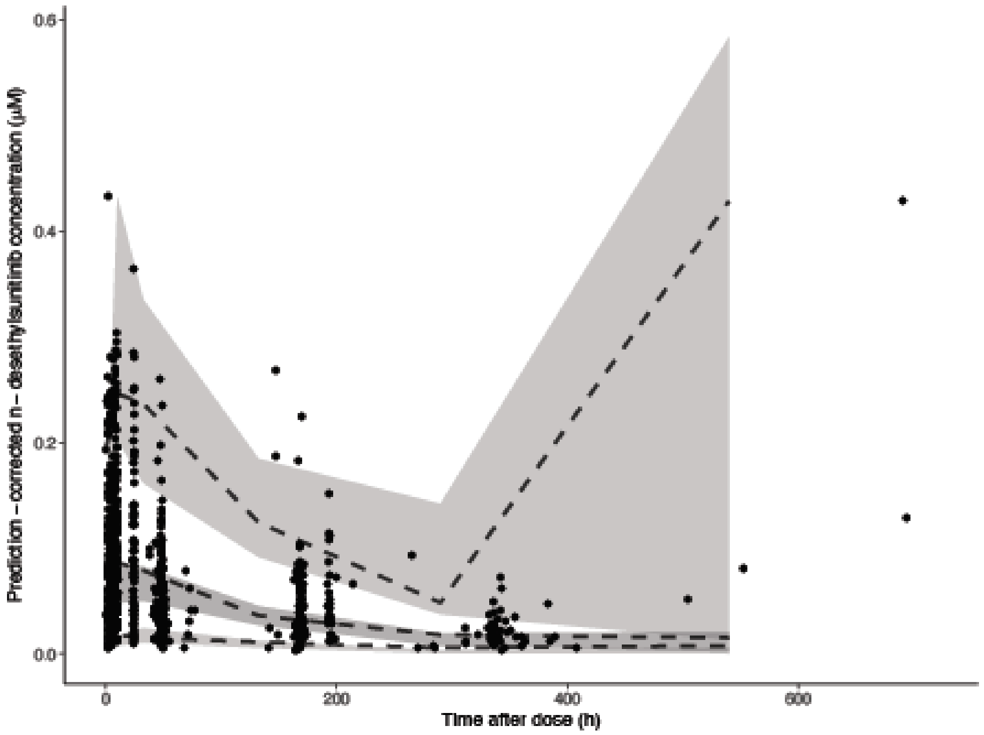

Supplement: Supplementary file 1 [file cancers-14-06061-s001.zip › cancers-2032617-SI/Supplementary Data S3.pdf]
