# Supplementary material for: High-Dose Intermittent Treatment with the Multikinase Inhibitor Sunitinib Leads to High Intra-Tumor Drug Exposure in Patients with Advanced Solid Tumors
Source: Cancers (Basel). 2022 Dec 9;14(24):6061. doi: 10.3390/cancers14246061 (PMC9775433; doi:10.3390/cancers14246061)
Supplement: Supplementary file 1 [file cancers-14-06061-s001.zip › cancers-2032617-SI/Supplementary Data S5.pdf]

Supplementary Data S5

Correlations between sunitinib + and N-desethyl sunitinib (SUM) tumor, plasma C_{max}, and skin concentration versus progression free survival (left) and overall survival (right)

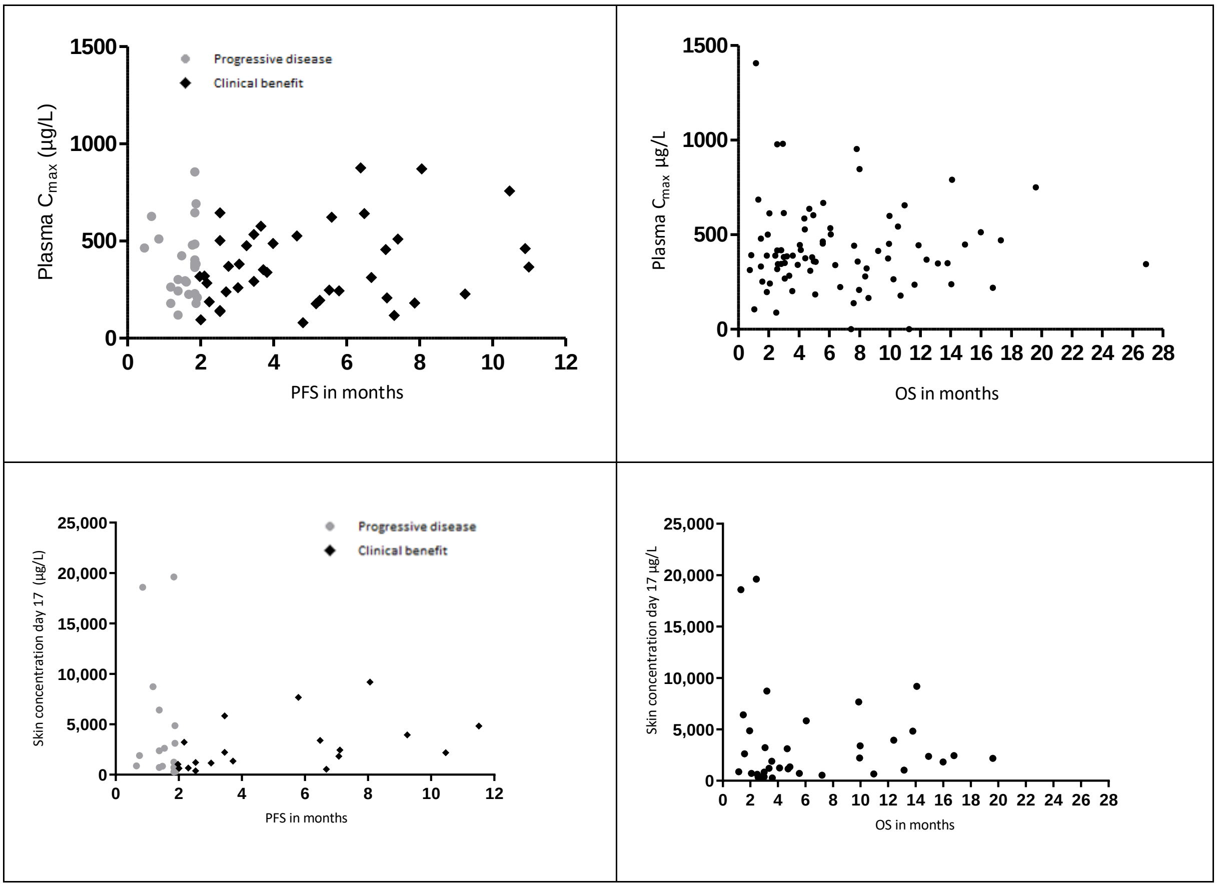

All concentrations represent the sunitinib + N-desethyl sunitinib sum concentration of an individual patient. The SUM concentration of sunitinib + N-desethyl sunitinib in tumor biopsies significantly correlated with PFS (Spearman's rank correlation coefficient (ρ) = 0.43, P-value 0.046) and OS (ρ 0.49; P-value 0.024) (Figure 3).

This was not the case for either plasma concentrations (Cmax (P-value for PFS 0.83, for OS 0.99), plasma Ctrough (P-value for PFS 0.19, for OS 0.42) and plasma Caverage (P-value for PFS 0.29, for OS 0.27) as well as the skin concentrations (P-value for PFS 0.48, for OS 0.63). Abbreviations; PFS, progression free survival; OS, overall survival, µg/L, microgram per Liter. A trendline has been added to the figures with a significant association.
