# Supplementary material for: High-Dose Intermittent Treatment with the Multikinase Inhibitor Sunitinib Leads to High Intra-Tumor Drug Exposure in Patients with Advanced Solid Tumors
Source: Cancers (Basel). 2022 Dec 9;14(24):6061. doi: 10.3390/cancers14246061 (PMC9775433; doi:10.3390/cancers14246061)
Supplement: Supplementary file 1 [file cancers-14-06061-s001.zip › cancers-2032617-SI/Supplementary Data S6.pdf]

Supplementary Data S6– Immunohistochemistry

No significant difference was observed for MVD pre- to on-treatment (semi quantification of the number of CD31+ vessels: pre-treatment 18 (range: 0-45) versus on-treatment 27 (range: 0-56) (paired *t*-test, mean increase 9.2, 95% CI -0.06 – 18.50, *P* 0.051).

In addition, no significant difference in tumor cell proliferation was found between the baseline and on-treatment biopsies (Ki67 ranged from 2% to 50% at baseline to 2% to 50% on-treatment (paired *t*-test, mean difference 3.11, 95% CI -2.68 – 9.08, *P*-value 0.26).
