# Supplementary material for: High-Dose Intermittent Treatment with the Multikinase Inhibitor Sunitinib Leads to High Intra-Tumor Drug Exposure in Patients with Advanced Solid Tumors
Source: Cancers (Basel). 2022 Dec 9;14(24):6061. doi: 10.3390/cancers14246061 (PMC9775433; doi:10.3390/cancers14246061)
Supplement: Supplementary file 1 [file cancers-14-06061-s001.zip › cancers-2032617-SI/Supplementary Data S7.pdf]

Kinome affinity profile of sunitinib using competition binding assays, parameter pKd, [15]

Target	µM
PDGFRB	0,0001
KIT(V559D,V654A)	0,0002
FLT3(K663Q)	0,0002
KIT(V559D,T670I)	0,0003
KIT	0,0004
FLT3	0,0004
KIT(V559D)	0,0004
PDGFRA	0,0008
FLT3(ITD)	0,001
DRAK1	0,001
KIT(L576P)	0,0013
FLT3-autoinhibited	0,0015
VEGFR2	0,0015
FLT1	0,0018
FLT3(D835Y)	0,0023
FLT3(N841I)	0,0024
CSF1R	0,0025
KIT-autoinhibited	0,003
CSF1R-autoinhibited	0,0037
FLT3(D835H)	0,0043
BIKE	0,0055
PHKG1	0,0055
RET(V804M)	0,0058
PHKG2	0,0059
RET(V804L)	0,0087
AXL	0,009
CHEK2	0,01
AAK1	0,011
FLT3(R834Q)	0,011
CSNK1E	0,013
ITK	0,013
RET	0,013
ULK2	0,013
IRAK1	0,014
CSNK1D	0,015
MYLK4	0,015
HPK1	0,016
PAK3	0,016
RSK3(Kin.Dom.1-N-terminal)	0,017
STK33	0,017
YSK4	0,017
AMPK-alpha1	0,019
LOK	0,019
MST1	0,019

RET(M918T)	0,019
CLK2	0,02
GAK	0,02
CLK1	0,022
DAPK3	0,022
MLCK	0,023
ULK1	0,023
TNIK	0,025
MERTK	0,026
RPS6KA5(Kin.Dom.1-N-terminal)	0,028
CLK4	0,029
MINK	0,029
HIPK2	0,031
MAP4K2	0,033
RIOK1	0,035
LKB1	0,038
PIP5K2B	0,039
HIPK3	0,041
MAP4K5	0,041
ULK3	0,042
KIT(A829P)	0,043
MEK5	0,046
ARK5	0,048
S6K1	0,048
JAK1(JH2domain-pseudokinase)	0,049
MYLK2	0,049
RIOK2	0,049
TYRO3	0,049
FLT4	0,05
ABL1(T315I)-phosphorylated	0,055
HIPK1	0,055
MST2	0,056
SLK	0,056
MAP3K2	0,057
SRPK3	0,059
MST3	0,063
TTK	0,063
BLK	0,065
IRAK4	0,066
ABL1(H396P)-nonphosphorylated	0,074
ABL1(Q252H)-phosphorylated	0,076
LRRK2(G2019S)	0,076
CAMK2A	0,08

PYK2	0,082
AMPK-alpha2	0,089
TAK1	0,093
LZK	0,095
RPS6KA4(Kin.Dom.1-N-terminal)	0,096
CSNK1A1	0,099
DLK	0,1
TRKA	0,1
CSNK1G2	0,11
DCAMKL3	0,11
DRAK2	0,11
KIT(D816H)	0,11
LRRK2	0,11
MEK2	0,11
ABL1(M351T)-phosphorylated	0,12
DAPK1	0,12
TBK1	0,12
YES	0,12
MEK1	0,13
ABL1(H396P)-phosphorylated	0,14
ABL1(Y253F)-phosphorylated	0,14
GRK4	0,14
MAP4K4	0,14
ROCK2	0,14
RSK1(Kin.Dom.1-N-terminal)	0,14
STK39	0,14
ABL1-phosphorylated	0,15
ABL1(T315I)-nonphosphorylated	0,15
DAPK2	0,15
PCTK1	0,15
SNARK	0,15
CSNK2A2	0,16
HIPK4	0,16
ALK	0,17
ABL1(E255K)-phosphorylated	0,18
GCN2(Kin.Dom.2,S808G)	0,18
GRK7	0,18
MAP4K3	0,18
PLK4	0,19
SRPK2	0,19
DYRK1A	0,2

MAST1	0,2
SBK1	0,2
TAOK3	0,21
AURKC	0,22
MAP3K3	0,22
SGK3	0,22
LCK	0,23
ABL1(Q252H)-nonphosphorylated	0,24
CSNK1G3	0,24
SRPK1	0,25
STK16	0,25
ABL1-nonphosphorylated	0,27
FGR	0,27
LYN	0,27
PFTK1	0,27
MYLK	0,28
PRKD3	0,28
FGFR3	0,29
GRK1	0,29
YSK1	0,29
CHEK1	0,3
ANKK1	0,31
MARK2	0,31
PRKD1	0,31
CDK7	0,33
TLK2	0,33
MST4	0,34
MELK	0,35
TYK2(JH2domain-pseudokinase)	0,36
ABL1(F317L)-phosphorylated	0,37
DCAMKL1	0,37
RIPK1	0,37
AURKB	0,38
KIT(D816V)	0,38
PRKD2	0,38
PRP4	0,39
JAK2(JH1domain-catalytic)	0,41
MARK3	0,41
NDR1	0,41
CAMK2D	0,42
CAMKK1	0,42
INSRR	0,43
CAMK1G	0,44

FAK	0,44
LATS2	0,46
ROCK1	0,46
ICK	0,47
EPHB1	0,48
MUSK	0,49
HUNK	0,5
INSR	0,5
CAMK1D	0,51
FGFR1	0,52
FYN	0,52
IKK-alpha	0,52
FRK	0,53
OSR1	0,53
CSNK1A1L	0,55
BMPR2	0,57
FGFR2	0,57
RSK2(Kin.Dom.1-N-terminal)	0,58
SIK2	0,58
TRKB	0,59
ERN1	0,6
IKK-epsilon	0,62
LATS1	0,63
PAK7	0,64
SNRK	0,64
PRKR	0,67
DYRK2	0,68
TNK1	0,68
CAMK2G	0,69
MEK4	0,7
PKN1	0,71
ABL1(F317L)-nonphosphorylated	0,73
TLK1	0,74
NIM1	0,85
EGFR(L858R,T790M)	0,86
HCK	0,88
ABL1(F317I)-phosphorylated	0,89
CAMK4	0,89
TAOK1	0,89
CSNK2A1	0,9
CSNK1G1	0,93
IRAK3	0,94
EPHA6	0,96
FES	0,96
CAMK1	0,97

NDR2	0,97
ABL2	1
EPHB6	1
BRSK2	1,1
CDKL2	1,1
FER	1,1
WEE1	1,1
EPHA5	1,2
JAK3(JH1domain-catalytic)	1,2
MARK1	1,2
MET(M1250T)	1,2
PCTK2	1,2
MAP3K15	1,3
MLK3	1,3
PKN2	1,3
RIPK5	1,3
STK35	1,3
CAMK2B	1,4
FGFR3(G697C)	1,4
NEK2	1,4
CAMKK2	1,5
TYK2(JH1domain-catalytic)	1,6
AURKA	1,7
MEK3	1,7
PCTK3	1,7
RPS6KA5(Kin.Dom.2-C-terminal)	1,7
LTK	1,8
SgK110	1,9
CDK4-cyclinD3	2
DDR1	2
RPS6KA4(Kin.Dom.2-C-terminal)	2
BTK	2,1
EPHA3	2,1
FGFR4	2,1
RIPK4	2,1
SRC	2,1
DYRK1B	2,3
PAK4	2,3
BMPR1B	2,4
EGFR(T790M)	2,4
EPHA7	2,4
JNK2	2,4
PAK6	2,4
PIM3	2,4

RSK4(Kin.Dom.1-N-terminal)	2,4
IGF1R	2,6
AKT2	2,7
DCAMKL2	2,7
DDR2	2,9
ACVR1	> 3
ACVR1B	> 3
ACVR2A	> 3
ACVR2B	> 3
ACVRL1	> 3
ADCK3	> 3
ADCK4	> 3
AKT1	> 3
AKT3	> 3
ASK1	> 3
ASK2	> 3
BMPR1A	> 3
BMX	> 3
BRAF	> 3
BRAF(V600E)	> 3
CASK	> 3
CDC2L1	> 3
CDC2L2	> 3
CDC2L5	> 3
CDK11	> 3
CDK2	> 3
CDK3	> 3
CDK8	> 3
CDK9	> 3
CDKL1	> 3
CDKL3	> 3
CDKL5	> 3
CLK3	> 3
CSK	> 3
DMPK	> 3
DMPK2	> 3
EGFR	> 3
EGFR(E746-A750del)	> 3
EGFR(G719S)	> 3
EGFR(L747-E749del, A750P)	> 3
EGFR(L747-S752del, P753S)	> 3
EGFR(L747-T751del,Sins)	> 3
EGFR(L858R)	> 3
EGFR(L861Q)	> 3
EGFR(S752-I759del)	> 3

EIF2AK1	> 3
EPHA1	> 3
EPHA2	> 3
EPHA4	> 3
EPHA8	> 3
EPHB2	> 3
EPHB3	> 3
ERBB2	> 3
ERBB3	> 3
ERBB4	> 3
ERK1	> 3
ERK2	> 3
ERK3	> 3
ERK4	> 3
ERK8	> 3
GSK3A	> 3
GSK3B	> 3
IKK-beta	> 3
JNK1	> 3
LIMK1	> 3
LIMK2	> 3
MAK	> 3
MAP3K1	> 3
MAPKAPK2	> 3
MAPKAPK5	> 3
MKK7	> 3
MLK2	> 3
MRCKA	> 3
MRCKB	> 3
MST1R	> 3
MTOR	> 3
NEK1	> 3
NEK11	> 3
NEK3	> 3
NEK4	> 3
NEK5	> 3
NEK6	> 3
NEK9	> 3
NLK	> 3
p38-alpha	> 3
p38-beta	> 3
p38-delta	> 3
p38-gamma	> 3
PAK1	> 3
PAK2	> 3
PFTAIRE2	> 3
PIK3C2B	> 3
PIK3C2G	> 3

PIK3CA	> 3
PIK3CA(C420R)	> 3
PIK3CA(E542K)	> 3
PIK3CA(E545A)	> 3
PIK3CA(E545K)	> 3
PIK3CA(H1047L)	> 3
PIK3CA(H1047Y)	> 3
PIK3CA(I800L)	> 3
PIK3CA(M1043I)	> 3
PIK3CA(Q546K)	> 3
PIK3CB	> 3
PIK3CD	> 3
PIK3CG	> 3
PIK4CB	> 3
PIM1	> 3
PIM2	> 3
PIP5K1C	> 3
PIP5K2C	> 3
PKAC-alpha	> 3
PKAC-beta	> 3
PKMYT1	> 3
PLK1	> 3
PLK3	> 3
PRKCD	> 3
PRKCE	> 3
PRKCH	> 3
PRKCI	> 3
PRKG1	> 3
PRKG2	> 3
PRKX	> 3
QSK	> 3
RAF1	> 3
RIPK2	> 3
ROS1	> 3
RSK4(Kin.Dom.2-C-terminal)	> 3
SRMS	> 3
STK36	> 3
SYK	> 3
TAOK2	> 3
TEC	> 3
TESK1	> 3
TGFBR1	> 3
TGFBR2	> 3
TIE2	> 3
TNNI3K	> 3
TRPM6	> 3
TXK	> 3

VRK2	> 3
WEE2	> 3
YANK1	> 3
YANK2	> 3
YANK3	> 3
ZAK	> 3
ZAP70	> 3
EPHB4	> 3
MYO3A	> 3
SIK	> 3
MLK1	> 3
BRSK1	> 3
PDPK1	> 3
ABL1(F317I)-nonphosphorylated	> 3
MARK4	> 3
RIOK3	> 3
CIT	> 3
MKNK1	> 3
TIE1	> 3
NEK7	> 3
JNK3	> 3
PRKCQ	> 3
MYO3B	> 3
BRK	> 3
MET(Y1235D)	> 3
PLK2	> 3
MAP3K4	> 3
RSK1(Kin.Dom.2-C-terminal)	> 3
TRKC	> 3
MEK6	> 3
PIP5K1A	> 3
MKNK2	> 3
JAK1(JH1domain-catalytic)	> 3
CDK4-cyclinD1	> 3
TSSK1B	> 3
EGFR(G719C)	> 3
CDK5	> 3
MET	> 3
CTK	> 3
RSK3(Kin.Dom.2-C-terminal)	> 3
TNK2	> 3
ERK5	> 3
