# Supplementary material for: High-Dose Intermittent Treatment with the Multikinase Inhibitor Sunitinib Leads to High Intra-Tumor Drug Exposure in Patients with Advanced Solid Tumors
Source: Cancers (Basel). 2022 Dec 9;14(24):6061. doi: 10.3390/cancers14246061 (PMC9775433; doi:10.3390/cancers14246061)
Supplement: Supplementary file 1 [file cancers-14-06061-s001.zip › cancers-2032617-SI/Supplementary Data S8.pdf]

Supplementary Data S8

Kinome affinity profile of sunitinib using affinity purification-mass spectrometry analysis, parameter IC_{50}.[15]

**Target**	**μM**
TNIK	0.37
MAP4K4	0.48
CLK1	0.48
RASSF3	0.8
FGFR-1	0.88
AAK1	0.88
TEX264	0.99
Q6ZSR9	1.03
PHKG2	1.03
MAP4K3	1.05
RASSF5	1.13
LATS1	1.30
CSNK1D	1.30
